# Rapid decline of renal function in patients with type 2 diabetes with heavy proteinuria: a report of three cases

**DOI:** 10.1186/s12882-019-1203-7

**Published:** 2019-01-16

**Authors:** Christopher Thiam Seong Lim, Nurul Zaynah Nordin, N. Z. Fadhlina, M. S. Anim, T. Kalaiselvam, W. Z. Haikal, Bak Leong Goh

**Affiliations:** 1 Unit of Nephrology, Department of Medicine, Universiti Putra Malaysia, Seri Kembangan, Malaysia; 2 Department of Nephrology, Serdang Hospital, 43400 Seri Kembangan, Selangor Malaysia; 3 Clinical Research Center, Serdang Hospital, 43400 Seri Kembangan, Selangor Malaysia; 4 Department of Medicine, Faculty of Medicine and Health Sciences, Universiti Putra Malaysia, Seri Kembangan, Selangor Malaysia

**Keywords:** Diabetes mellitus, Rapid decliners, Risk factors, Proteinuria, Tubulointerstitial inflammation traditional medications

## Abstract

**Background:**

Although there is a large volume of literature regarding the definition and epidemiology of.

Type 2 diabetes nephropathy (T2DN). There has been a paucity of data focused on the rate of transition of T2 DN. Based on our personal observation a certain percentage of our incident end stage renal disease (ESRD) patients from T2DN experienced a rapid decline of renal function. Their rapid decline nature of glomerular filtration rate (GFR) of 46 to 60 mL/min per 1.73m^2^ per year have far exceeded the KDIGO definitions of acute kidney injury (abrupt decrease in kidney function occurring over 7 days or less), acute kidney disease (acute or subacute damage and/or loss of kidney function for a duration of between 7 and 90 days after exposure to an acute kidney injury initiating event (Chawla et al Nat Rev Nephrol 241–57 2017) or even rapid decliner (eGFR declines > 5 mL/min per 1.73m^2^ per year) (Chawla et al Nat Rev Nephrol 241–57 2017; Andrassy Kidney Int 622–623 2013).

**Case presentation:**

We describe here three cases of type 2 diabetic patients that have rapid renal deterioration with rate of decline 46 - 60 mL/min per 1.73m^2^ per year. All the patients are heavily nephrotic. All of the renal biopsies done showed the classical diabetic changes, hypertensive changes, diffuse tubulointerstitial damage, and interstitial nephritis. All of the patients admitted to taking various form of traditional medications in hope of curing their renal disease.

**Conclusion:**

We wish to highlight that type 2 diabetics with massive nephrotic range proteinuria have enhanced risk of rapid renal function deterioration. The patients should be educated about the risks of rapid renal function deterioration when there is presence of heavy proteinuria. High grade proteinuria is likely to inflict the diffuse tubulointerstitial inflammation. The interstitial nephritis could be further worsened by traditional supplements consumption. Timely health education and advice must be undertaken to retard this unwanted rapid renal disease progression.

## Background

Type 2 Diabetes Mellitus (T2DM) is a major non-communicable disease in Malaysia with diabetic nephropathy (DN) leading as the main etiology of end stage renal disease (ESRD). The incidence of ESRD secondary to DN is fast increasing over the years. This is mainly due to increasing sedentary lifestyle in our patients. Effective detection and early intervention in DN can help to slow renal function decline. It can also prevent complications, thus improving survival and quality of life in type 2 diabetics. Annual decline in glomerular filtration rate (GFR) in a person varies widely depending on various factors such as ethnicity, age, underlying medical problems, the etiology of chronic kidney disease (CKD), and the presence of comorbidities. Kidney Disease: Improving Global Outcomes (KDIGO) guidelines define rapid progression as rate of eGFR declines > 5 mL/min per 1.73m^2^ per year [1, 2]. The classical progression of DN is deterioration of renal function over decades with typical rate of GFR decline ranges from between 2 to 20 mL/min per 1.73m^2^ per year with a median of 12 mL/min per year [3]. In our report, heavy amount of proteinuria, beside the traditional risk factors like poor glycemic control, hypertension are the leading risk factors for rapid progression of renal disease.

## Case presentation

### Case 1

**Fig. 1:**
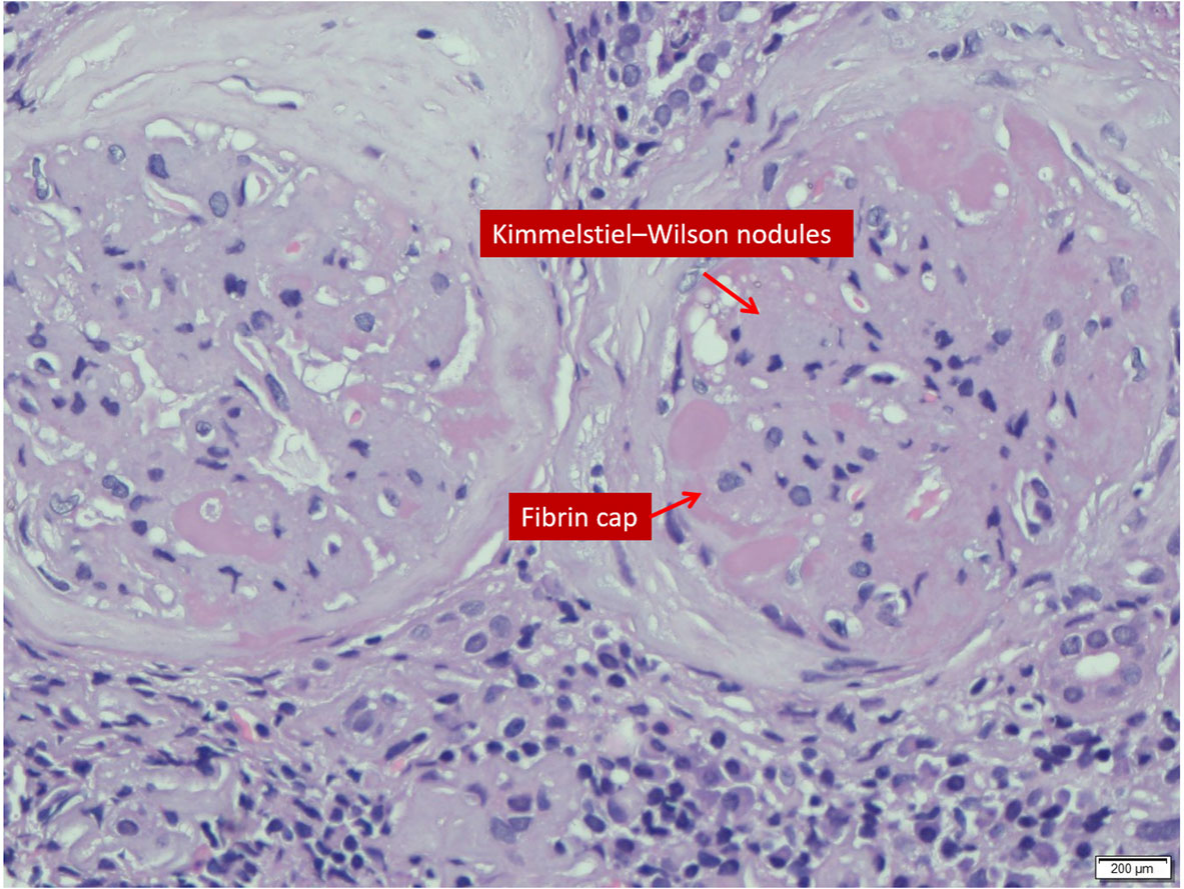
Kimmelstiel-Wilson nodules

Madam A, 45-year-old Malay lady diagnosed T2DM since age 30 years old, however has never been compliance to treatment. Since 2011 she has been suffering from frequent infections occurring at buccal space, left hand and right foot, requiring antibiotics, incision and drainages. Her glucose control was poor with Hba1c ranged around 12% with serum creatinine ranging around 60–70 μmol/l with proteinuria 4+. She was first seen in Nephrology clinic in March 2015 when she presented with bilateral lower limb swelling and deranged renal profile. Her serum creatinine was 120 μmol/l (eGFR 65 mL/min per 1.73m^2^) that time with low albumin 28 g/L and persistent nephrotic range proteinuria with urine protein-creatinine index (UPCI) at 0.23–0.3 g/mmol. Ophthalmology review noted bilateral moderate non-proliferative diabetic retinopathy. She was treated with basal bolus insulin, diuretic and statin but subsequently defaulted nephrology follow-up. Patient was referred again from the health clinic a year after (March 2016) for similar complain but this time with much worsening renal function. Her creatinine this time was 222 μmol/l and her medications at this time was amlodipine 10 mg daily, hydrochlorothiazide 25 mg daily, subcutaneous (SC) isophane insulin 6 IU BD and simvastatin 40 mg ON. Patient again did not turn up for her subsequent follow up and admitted on trying alternative medicine for her renal impairment. She came again in August 2016 presented with apparent nephrotic syndrome with grossly edematous lower limb with ascites and poorly controlled blood pressure to the emergency department. Her blood investigations showed serum creatinine 612 μmol/l (eGFR 12 mL/min per 1.73m^2^), UPCI 1.48 g/mmol, urinalysis: blood 1+/ protein 4+/ leukocytes negative (Table 1). Urgent renal doppler ultrasound showed normal size kidneys, no evidence of obstructive uropathy or renal vein thrombosis. In view of sudden drop in renal function a renal biopsy was planned and results showed features of advance diabetic nephropathy, modular glomerulosclerosis pattern with 60% global sclerosis and moderate hypertensive vascular changes associated with severe chronic tubulointerstitial damage. There are also features of interstitial nephritis seen. Diabetic nephropathy (Tervaert Class IV) with moderate hypertensive vascular changes and interstitial nephritis (Fig. 1). She was counselled on long term renal replacement therapy and she has been dialysis dependant since then.

**Table 1:** Madam A - Laboratory and clinical measurements

	March 2015	March 2016	Aug 2016
Creatinine (μmol/L)	120	222	612
GFR (mL/min)	65	26	11
Hbaic %		6.2	
Proteinuria (g/day)	2.2	14.8	26.9
BP (mmHg)	120/70	146/82	161/65
Weight	53.7	58	68

### Case 2

**Fig. 2:**
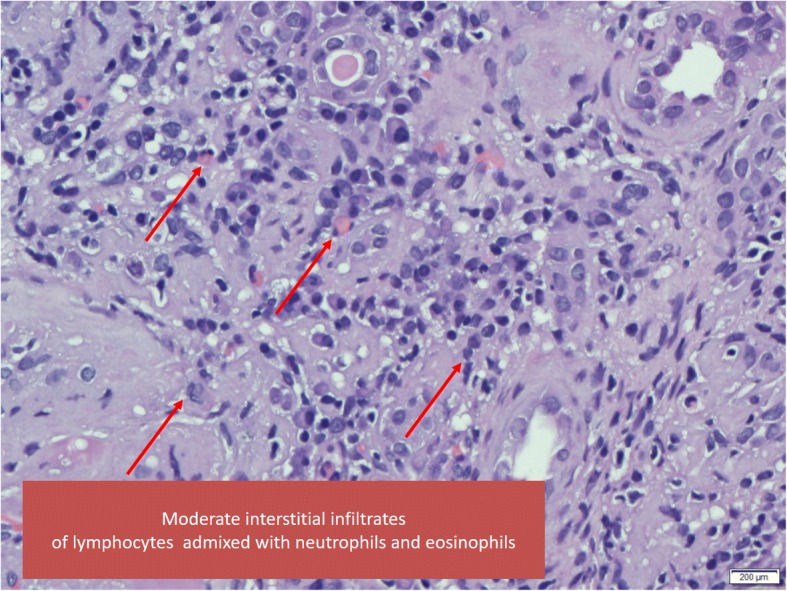
Tubulo-interstitial inflammation

Mr. B, 41 Malay male who was newly diagnosed T2DM and Hypertension about a year ago in a local private hospital. His initial presentation was nephrotic syndrome with normal renal function. He was initiated on subcutaneous premixed isophane insulin 20 IU BD, amlodipine 10 mg daily, perindopril 8 mg daily and lovastatin 20 mg ON. Patient was however non-compliance to his medications and went to seek traditional medications and also alternative medications. He presented to our hospital in May 2016 for worsening bilateral lower limb swelling associated with on and off breathlessness for 2 months. On examination he has bilateral pleural effusion, pedal edema and ascites. He was hypertensive with BP 170/100 mmHg. His baseline creatinine done 2 months earlier was 183 μmol/l. During this current presentation blood investigations noted creatinine of 359 μmol/l (eGFR 33 mL/min per 1.73m^2^), albumin 16 g/l, Hba1c 7.4% and UPCI 1.87 g/mmol He was treated with intravenous diuresis and upon discharge was advised for fluid and diet control. Mr. B was admitted again with gross bilateral lower limb swelling and right forearm abscess in July 2016. During this admission he was treated as acute on chronic kidney disease secondary to right forearm abscess. His admission creatinine was 481umol/l and it peaked to 947 μmol/l (Table 2). His renal function did not recover despite antibiotic and dialysis support. Renal ultrasound was normal. Renal biopsy was performed and it was reported as diabetic nephropathy (Tervaert Class III) with moderate to severe hypertensive vascular changes and interstitial nephritis (Fig. 2). Patient became dialysis dependant and currently is on hemodialysis as his mode of long term RRT.

**Table 2:** Mr. B - Laboratory and clinical measurements

	March 2016	May 2016	July 2016
Creatinine (μmol/L)	183	359	947
GFR (mL/min)	60	33	12
Hbaic %	6.9	7.4	–
Proteinuria (g/day)	–	18.7	–
BP (mmHg)	179/97	151/103	192/112
Weight (kg)	85	97.4	92.2

### Case 3

Madam C, 33-year-old Malay lady, diagnosed with T2DM since 20-year-old and hypertension at the age of 30-year-old. She was previously under health clinic follow up but was not compliance to her medications including oral hypoglycemic agents. Her Hba1c in 2015 was 11.9% and she was started on basal bolus insulin since a year ago. Her medications consisted of SC short acting insulin 26 IU tds, SC isophane insulin 26 IU on, metformin 1 g bd, amlodipine 10 mg daily, perindopril 4 mg daily and hydrochlorothiazide 12.5 mg daily. Her baseline renal function in April 2015 was normal with serum creatinine 95 μmol/l. During follow up, her BP was always in suboptimal range, and urinalysis showed persistent proteinuria 4+ with glucosuria. She was referred to nephrology clinic in august 2016 for rapid decline in renal function and worsening pedal edema. On examination noted she was hypertensive with BP 180/100 mmHg and bilateral lower limb edema up to the thighs.

Her baseline serum creatinine trend was 165 μmol/l with eGFR 54 ml/min in November 2015. This has deteriorated to 366 μmol/l (eGFR 24 mL/min per 1.73m^2^) in May 2016 and in August 2016 her renal function further deteriorated with serum creatinine of 557 μmol/l (eGFR 16 mL/min per 1.73m^2^). Urinalysis showed protein 4+/ Blood +/−, Glucose 4+, Leucocytes negative. Her serum was albumin 25 g/L and Hba1c 10.2%, Anti-nuclear antibody was no-reactive, and her urine protein creatinine index was 1.38 g/mmol (Table 3). Ultrasound kidneys showed normal kidney sizes with no obstructive uropathy. Ophthalmology review noted bilateral extensive proliferative diabetic retinopathy. She has admitted to have tried traditional medications. She was counselled for renal biopsy in view of sudden drop in renal function and hemodialysis had to be initiated. Renal biopsy showed Diabetic nephropathy (Tervaert Class III) with moderate to severe hypertensive vascular changes and tubulointerstitial nephritis.

**Table 3:** Madam C - Laboratory and clinical measurements

	April 2015	May 2016	Aug 2016
Creatinine (μmol/L)	95	165	557
GFR (mL/min)		54	16
Hbaic %	11.9	–	10.2
Proteinuria (g/day)	–	–	13.8
BP (mmHg)	150/86	180/100	160/96
Weight (kg)	70	77	

## Discussion and conclusion

Diabetic nephropathy (DN) or diabetic kidney disease is characterized by its unique functional and structural changes. During the initial phase of clinically silent period, important structural changes occur. These include glomerular basement membrane thickening, mesangial proliferation, podocyte injury, and glomerular sclerosis. These histopathologic abnormalities are noted to be presence before the onset of moderately increased albuminuria. On the other hand, functional changes include hyperfiltration, micro and macroalbuminuria, with incipient progressive proteinuria that slowly progressed into chronic kidney disease and eventually ESRD.

In a study of carried out in San Francisco outpatient dialysis units, 7.6 to 12.7% of the patients were noted to have suffered from rapid declined in kidney function prior to the initiation of permanent dialysis [4]. Unfortunately, the author did not provide much information about causes of the rapid declined in kidney function or any remedial step was taken. The definition of “rapid decline in kidney function” was also arbitrary [4]. In a logistic regression model with rapid decline as the outcome, the Spanish researchers has reported that previous cardiovascular disease and higher proteinuria were the main predictors of rapid kidney decline (odd ratio 1.8–1.9) [5]. In the same study, the rapid decliners consisted of more diabetic patients. In the Cardiovascular Health Study done by Shlipak et al. which included 4380 individuals older than 65 years of age, the rapid kidney function decliner group are mainly made up of diabetic patients [6].

We summarized here 3 cases of T2DM patients with rapidly declining renal function (46 to 60 mL/min/year) and all had undergone renal biopsies for unexplained reduction in eGFR (Table 4). All three patients were noted to have overt proteinuria at diagnosis. Only one patient had respectable Hba1c at 7.4% while the rest had poorly controlled diabetes with Hba1c ranged more than 10%. However, it is important to note that the patient with better Hba1c had anemia at presentation and this may have led to a falsely low A1c reading. All patients had poorly controlled BP during follow up, and 2 patients had acute kidney injuries secondary to infections that have accelerated the renal deterioration. 2 patients admitted taking alternative treatment for diabetes mellitus beside being non-compliant to their diabetic medications. Their renal biopsies showed diabetic nephropathy with chronic, severe tubulointerstitial damage and interstitial nephritis (Figs. 1 and 2).

**Table 4:** Summary of the patients’ data

	Madam A	Mr B	Madam C
Gender	Female	Male	Female
Ethnicity	Female	Malay	Malay
Age	45	41	33
Diabetes (Years)	15	< 1 year	12
Hypertension (Year)	1	1	3
Drop in GFR in mL/min/year	46	46	60 mL
Renal ultrasound	Normal	Increased parenchymal echogenicity	Normal
Renal biopsy	Diabetic nephropathy, hypertensive changes with interstitial nephritis	Diabetic nephropathy, hypertensive changes with interstitial nephritis	Diabetic nephropathy, hypertensive changes and interstitial nephritis
Hbaic (%)	6.2	7.4	10.2
Albumin (g/L)	24	12	29
Proteinuria g/day	14.8	18.7	13.8
Tried supplements	Yes	Yes	Yes

Proteinuria has long been recognized as an independent risk factor for renal function loss. It is the cardinal feature of acute and chronic kidney disease. The presence and severity of proteinuria has been shown to be a reliable strategy to identify rapid renal decline in community-based prospective cohort study [7]. Measurement of total protein in urine is an inexpensive and well-established marker for kidney injury. The failing kidney is characterized histologically by tubulointerstitial inflammation, tubular cell apoptosis, tubular atrophy and fibrosis, and these changes correlate with the severity of proteinuria. Heavy tubular proteinuria also predicts a longer duration of interim dialysis support in patient with kidney injury in our population [8].

In healthy kidneys, proximal tubules epithelial cells (PTEC) can reclaim proteins that have managed to pass the glomerular filtration barrier via endocytosis. The complex mechanisms of PTEC protein handling involve the much studied megalin-cubilin complex [9]. However, this process is compromised in diseased glomeruli, where excess filtration of bioactive proteins into the tubular fluid can dysregulated PTEC signaling pathways in response to protein leakage from the glomeruli. As a consequence, there are abnormal PTEC growth, apoptosis, gene transcription and inflammatory cytokine production [9].

Traditional supplement has been known to contain various herbal compounds that are nephrotoxic to kidneys. Aristolochic acids, anthraquinones, flavonoids, and glycosides from herbs have all been implicated for causing nephropathy. The exact pathogenesis of supplement related AKI has not been well established. Most of the time, the diagnosis is made via history of consumption of the supplement and demonstration of acute tubular necrosis and acute interstitial nephritis as per our case [10]. It has been our experience that the renal functions of patients who have heavy proteinuria tends to suffer the most when they also taking herbal compounds concurrently. The magnitude of deterioration is as high as 60 mL/min per year in our case series.

Two of the patients (Madam A and Mr. B) have recurrent infections and needed courses of antibiotics. None of them have received non-steroidal anti-inflammatory drugs or contrast agents. However, all of them did receive diuretics. The prescribed medicines and the rapid fluctuation in their extracellular water compositions may have inadvertently cause some degree of interstitial nephritis or pre-renal acute kidney injuries.

DN patients are the one of the most common nephropathies being referred to our instituation [11]. In our experience, patients who has proteinuria of more than 3 g per day do experience rapid deterioration of renal function over years, however the magnitude of deterioration will be less than 10 ml/min/year. It was the rapid deterioration of the renal function that has prompted to perform diagnostic renal biopsies.

There are many ways to retard diabetic nephropathy progression. Current management practices advocate that the best way to reduce microvascular and macrovascular complications is by treating the sugar control to target. In addition, as the rate of progression DN is also closely related to blood pressure control at baseline, a good blood pressure control plays an equally important role in retarding DN progression. As a matter of fact, it has been demonstrated that for DN patient with a background of hypertension, glycemic control may have little independent predictive value [12]. This is further strengthened by the findings from the United Kingdom Diabetes Prospective Study (UKPDS), whereby a sustained reduction in blood pressure came out as the most important single intervention that could slow down the progression of diabetic nephropathy in both Type 1 and type 2 DM patients [13].

Current guideline suggests a blood pressure goal of less than 140/90 mmHg in T2 DM patients and in addition it emphasis on good control of glycemia, obesity management, lowering lipids and cessation of smoking. Achieving this target is also associated with renoprotective effects, although there may be particular advantages conferred following blockade of the renin- angiotensin system (RAS) [14]. Novel treatment strategies targeting different pathway, such as inflammation, fibrosis, and oxidative stress— are on-going for treatment of DN [15].

Data from the Japan Diabetes Complications Study (JDCS) showed that in normo- and micro albuminuric patients with type 2 diabetes, patients with hyperfiltration (eGFR ≥120 ml/min/1.73 m2) have high risk of rapid renal function deterioration [16]. It is also important to retard the microalbuminuria as once overt proteinuria develop, it is almost difficult to retard the CKD progression.

Currently there is no one-off investigations that can distinguishing fast decliners from slow decliners. Serial measurements of serum creatinine performed in the clinic often detect late stages of the fast decliners. The traditional end points used in clinical trials such as doubling of serum creatinine level or ESRD are not suitable to detect rapid decliners and this has hamper the effective evaluation of clinical trials in rapid decliners. A more suitable outcome measurement should include the rate of annual renal function declined over 2 to 3 years. Massive interest is underway to find an effective prognostic marker that identifies decliners at a single clinical encounter. Until recently 30 biomarkers showed significant associations with rapid progression after adjusted for clinical characteristics. Some of the well-known biomarkers identified are β2-microglobulin, Kidney injury molecule-1 and cystatin-C, whereas others have little or no prior data (for example, Symmetric dimethylarginine/asymmetric dimethylarginine ratio, Fibroblast growth factor-21 and uracil) [17]. Elevated serum TNF receptor 1 and 2 have shown early promise to become effective predictors of fast renal decline to ESRD in diabetic population [18].

Understanding the mechanisms underlying heavy proteinuria in T2DN that leads to rapid renal deterioration is desired to halt this undesirable event. Future studies will include identifying bio-markers, understanding individual variation in rate of decline, designing novel treatment targets and perhaps individualizing treatment for T2DN.

## Abbreviations

CKD: Chronic kidney disease
DM: Diabetes mellitus
DN: Diabetic nephropathy
ESRD: End stage renal disease
GFR: Glomerular filtration rate
PCI: Urine protein creatinine index
PTEC: Proximal tubules epithelial cells
